# Retraction Note: Computational Drug Repositioning for Gastric Cancer using Reversal Gene Expression Profiles

**DOI:** 10.1038/s41598-022-13460-2

**Published:** 2022-06-13

**Authors:** In-Wha Kim, Hayoung Jang, Jae Hyun Kim, Myeong Gyu Kim, Sangsoo Kim, Jung Mi Oh

**Affiliations:** 1 College of Pharmacy and Research Institute of Pharmaceutical Sciences, Seoul National University, Seoul, Republic of Korea; 2 Graduate School of Clinical Pharmacy, CHA University, Pocheon, Republic of Korea; 3 Department of Bioinformatics and Life Science, Soongsil University, Seoul, Republic of Korea

Retraction of: *Scientific Reports* 10.1038/s41598-019-39228-9, published online 25 February 2019

The authors of this Article have retracted it for failing to appropriately cite and acknowledge prior work by Chen et al.^1^. The Article uses methodology reported by Chen et al. to identify candidate drugs for gastric cancer.

All authors agree with this retraction.
